# Functionalization of the Surface of Porous Nickel–Titanium Alloy with Macrocyclic Compounds

**DOI:** 10.3390/ma16010066

**Published:** 2022-12-21

**Authors:** Ekaterina Marchenko, Venera Luchsheva, Gulsharat Baigonakova, Abdigali Bakibaev, Alexander Vorozhtsov

**Affiliations:** Laboratory of Superelastic Biointerfaces, National Research Tomsk State University, 36 Lenin Ave., 634045 Tomsk, Russia

**Keywords:** TiNi, porosity, bambusuril, supramolecular compounds surface, cytocompatibility, functionalization, antibacterial effect

## Abstract

For the first time, we performed functionalization of the surface of porous titanium nickelide alloys with bambusuril[6]-based macrocyclic compounds by different methods in order to provide the basis for saturation with therapeutic agents to impart antibacterial activity and accelerate its osteogenesis. It has been shown for the first time that the vacuum modification method is preferable for bambusuril deposition, since it provides a uniform deposition of organic matter on both the outer and inner surfaces of the pores. The effect of bambusuril deposition methods on the continuity, structure, and cytocompatibility of the porous titanium nickelide surface was evaluated. In vitro tests proved high biocompatibility and low toxicity of porous TiNi treated with BU[6] under vacuum. The SEM study of the structure of the surface layer of TiNi modified with BU[6] under the vacuum method showed that BU[6] agglomerates are uniformly deposited on the inner and outer surfaces of TiNi pores, which will provide an even saturation of BU[6] cavities with various pharmaceuticals, including antibiotics and inhibitors.

## 1. Introduction

Porous titanium nickelide (TiNi) alloys are currently widely used in the field of bone tissue replacement and regeneration. The structure of these alloys is porous permeable, similar to the structure of bone tissues; such alloys can long withstand physiological loads in the body and retain the frame function for bone tissue ingrowth [1,2,3,4]. Excellent functional properties of these alloys cannot prevent postoperative infectious complications, which does not allow the newly formed bone tissue to fill the formed spaces in a timely way and hinders patient rehabilitation. To minimize these effects, biocompatible materials should simultaneously perform the function of a long-term functioning implant and of a bone substitute carrier saturated with drugs (inhibitors of inflammation, antibiotics).

Well-known methods of surface functionalization are biochemical methods of TiNi modification, which are based on the covalent interaction of silanized titanium with peptides, proteins, or growth factors [5,6,7,8]. However, a number of limitations of these biochemical methods include long-lasting titanium silanization and subsequent immobilization of bioactive compounds, low cell proliferation, short shelf life, and stability of implant proteins [9]. In addition, proteins can cause adverse immune responses [10]. As a result, all of the above methods make it very difficult to achieve the desired long-lasting effect.

Methods used to modify the surface by saturation with bioactive compounds, in particular supramolecular compounds, for controlled release of antibiotics, drugs, bioactive agents, and cells, are currently gaining relevance [11]. Supramolecular compounds are often more advantageous than other drug delivery systems, such as dendrimers, liposomes, micelles, carbon nanotubes, and hydrogels, as well as polymers, which are limited in stability and do not control the drug release rate [12,13,14,15,16]. It should be noted that supramolecular compounds can deliver drugs at the desired target and protect drug molecules from degradation [17,18].

Appropriate initial reagents for this synthesis are bambusuril-based supramolecular systems—a family of macrocyclic compounds that consist of n-2,4-substituted glycoluryl units connected through a row of n-methylene bridges along the equator of the macrocycle [17,18,19]. These macrocycles combine structural features of both cucurbiturils[n] and hemicucurbiturils[n] [19]. The Bambusuril[6] (BU[6]) cavity is about 12.7 A, which is a significantly deeper cavity than that of cucurbit[6]uril, which is 9.1 $\dot{A}$. These macrocyclic molecules are capable of encapsulating therapeutic agents through formation of supramolecular systems, releasing them non-covalently and sustainably under the impact of various factors (hV, pH, T). BU[6] binds anions within its hydrophobic cavity via 12 weak C-H•••A- hydrogen bonds [19]. The electrostatic surface potential indicates the BU[6] cavity as the most electropositive region, which therefore attracts anions, while the terminal carbonyl oxygen atoms form the most negative region, which in turn can be attracted to positively charged particles. Several factors contribute to the exceptional binding of bambusurils in solution. One is the stabilization of the anion inside the cavity by multiple hydrogen bonds CH•••A. The next driver of binding is the non-classical hydrophobic effect. The non-classical hydrophobic effect is an enthalpy-driven process that occurs as a result of the release of so-called high-energy water molecules from the host cavity upon guest complexation and their incorporation to a water molecule network in solvent bulk [20]. The enthalpy factor partially compensated by unfavorable entropy has been observed for anion binding inside bambusuril[6] [21].

Selective functionalization of bambusurils will allow them to be used for different purposes such as anion transport [19,22], diagnosis and treatment, modification of stationary phases of chromatography columns [23], transmembrane transport of Cl^−^ and HCO_3_^−^ anions through lipid bilayers [24], and others. New materials based on methyl viologen and BU[6] can be used to develop energy storage systems and light emitting diodes [24]. BU[6] can also be used as a carrier in liquid membranes for electromembrane extraction [25]. Chiral bambusurils bind with ibuprofen, N-acetylphenylalanine, N-acetylleucine, mandelic acid, and α-methoxyphenylaceticacid [25].

Currently, there is no literature about the study of BU[6] cytotoxicity; however, we can assume that BU[6] is safe for an organism because BU[6] is an analog of CB[6] which does not reduce the viability of cells at a concentration of 1 mM and below [26]. Concentrations of BU[6] above 0.9 mM may not be required for saturation of TiNi alloys. Furthermore, there is no information about the use of bambusurils for saturation of different scaffolds. Nowadays, only cucurbit[6]uril-anchored silica gel is synthesized via reaction of perallyloxycucurbit[6]uril and mercaptopropyl-functionalized silica gel [27]. The chemistry of bambusurils requires further study. Some BU[6] research is currently under development in our group.

There have been no works where a macrocyclic compound was used for bioactive molecules that fill TiNi porous materials to increase biocompatibility and saturate with antibacterial agents. The only well-known methods of surface functionalization are the biochemical methods of TiNi modification, which are based on the covalent interaction of silanized titanium with peptides, proteins [5,6,7,8]. However, peptides have low stability. Especially, they are stable only at 37 °C for one week. Also, peptides can stimulate unwanted immune responses in the body [10]. In addition, not long ago, research was published on renewable antibacterial porous polymeric coatings to enable titanium biomaterials to prevent and treat peri-implant infection [28]. Scientists constructed a porous N-halamine polymeric coating on a titanium surface through surface pore-making, surface grafting, and N-Cl functionalization. Surface pore-making via alkali-heat treatment provides the titanium surface with a well-developed porosity and high surface area. However, the deposition of bambusuril[6] does not require functionalization of the TiNi surface via alkali-heat treatment. In this work, bambusuril[6] was deposited directly on the surface of a porous material without covalent interaction.

In this study, BU[6] has been deposited on the surface of porous TiNi by different methods for the first time. This development will provide the basis for saturating the BU[6] cavity with therapeutic agents to impart antibacterial activity to titanium nickelide and accelerate its osteogenesis. Therefore, the purpose of this study is to evaluate the effect of BU[6] deposition methods on the continuity, structure, and in vitro cytocompatibility of the porous TiNi surface.

## 2. Materials and Methods

### 2.1. Preparation and Functionalization of the Surface of Porous TiNi

Porous TiNi alloys were obtained by self-propagating high-temperature synthesis (SHS) [3]. SHS was performed in a continuous layer-by-layer combustion mode in a flow reactor under an argon atmosphere. A powder mixture of 50 at.% Ti and 50 at.% Ni was poured into a quartz tube and then placed into the reactor. Synthesis was initiated at 430 °C. Flowing argon was fed into the reactor at a pressure of 0.01–0.05 MPa. The porosity of the obtained alloys was 60–65.5%. The dimensions of the porous samples were 5 × 5 × 2 mm^3^.

BU[6] synthesis. 2,4-Dimethylglycoluril (9 g, 53 mmol) and paraformaldehyde (1.6 g, 53 mmol) were heated at 40 °C in HCl (5.4 M, 30 mL) until the starting material had dissolved completely. The solution was then allowed to cool down and was stirred at room temperature for 24 h. The resulting precipitate was collected by filtration, washed with concentrated HCl and water, and dried under vacuum to give BU[6] as a white solid in 30% yield. Ref. [29] M.p. > 300 °C (dec.); ^1^H NMR (400 MHz, [D6]DMSO/CHCl_3_ (1:1), 30 °C, TMS), ppm: 5.29 (s, 12H), 5.06 (s, 12H), 2.51 (s, 36H). ^13^C NMR (100.63 MHz, [D6]DMSO/CDCl_3_ (1:1), 30 °C, TMS), ppm: 159.32, 158.45, 67.82, 48.78, 31.06.

BU[6] deposition on porous TiNi samples. The working solution of BU[6] for TiNi modification was prepared by dilution of 0.517 g of BU[6] in 150 mL mixture of CHCl_3_:DMSO (1:1). C (solution) was 0.317 g/mol. All TiNi samples were round in shape with diameter 1 cm. Prior to modification, TiNi samples were washed in an Elmasonic P30H ultrasonic bath (ultrasound power of 37 kHz) in distilled water for 30 min and then dried in air. Functionalization of the porous TiNi surface was performed by modification methods which employed effects of vacuum, microwaves, and ultrasound, and modification without external effects. Vacuum modification method: the sample was placed on a Schott funnel, and the working solution was poured under vacuum (vacuum range 102 Pa) for 15 min. After modification, the sample was washed with distilled water and dried in the LT-VO/50 vacuum oven for 10 h. Microwave modification method: the sample was placed in an autoclave, 15 mL of the working solution was added, and then it was transferred to the Speedwavefour microwave system. The modification parameters were as follows: power of 1160 W, temperature of 50 °C, duration of 40 min. After modification, the samples were stored in the working solution for 24 h. Then they were washed with distilled water and dried in the LT-VO/50 vacuum oven for 10 h. Ultrasound modification method: the sample was placed in a flask, and 15 mL of BU[6] solution was added. Next, the flask was placed in the Elmasonic P30H ultrasonic bath (ultrasound power of 37 kHz) for 40 min at room temperature. After modification, the sample was stored in the working solution for 24 h. Then it was washed with distilled water and dried in the LT-VO/50 vacuum oven for 10 h. Modification method without external effects: a TiNi sample was placed in a bottle and 15 mL of BU[6] solution was added. The sample was stored in the working solution for 24 h. After that, it was washed with distilled water and dried in the LT-VO/50 vacuum oven for 10 h.

### 2.2. Surface Characterization Methods

The samples’ structures were studied by scanning electron microscopy (SEM, VEGA 3 SBH, Tescan, Brno, Czech Republic). Energy dispersive X-ray spectroscopy (EDS, AztecLive Lite Xplore 30, Oxford Instruments, Abingdon, UK) was used for elemental analysis.

NMR spectroscopy analysis was carried out using a Bruker AVANCE 400 III HD NMR spectrometer (Bruker, Billerica, MA, USA). One-dimensional spectra were recorded for the nuclei of ^1^H atoms (frequency of 400.17 MHz) and ^13^C (frequency of 100.63 MHz) to confirm the BU[6] structure. Dimethylsulfoxide (DMSO D-6) with 99.9% atom D and heavy water (D_2_O) were used as solvents.

Thermogravimetric analysis (TGA) was performed using a TG–DTA Instrument (NETZSCH STA 449F1). Approximately 5 mg of the sample was weighed and heated from room temperature to 600 °C at a heating rate of 10 °C/min under a nitrogen flow rate of 20 mL/min.

The cytocompatibility was analyzed using the standard MCF-7 cell line (Purchased from Collection of Vertebrate Cell Cultures, Institute of Cytology, Russian Academy of Sciences. St. Petersburg 194064, Tikhoretsky pr. 4), which is most widely used for in vitro studies of the pharmaceutical cytotoxicity and cytocompatibility of various biocompatible materials [30]. For the study, the modified TiNi samples were pre-sterilized for 60 min at 120 °C in a dry oven. MCF-7 cells were cultured in a CO_2_ incubator for 24 h under standard conditions at 37 °C, in a humidified atmosphere of 5% CO_2_. The culture medium comprised DMEM/F12 (Paneco, Moscow, Russia) supplemented with 10% fetal bovine serum (Paneco, Moscow, Russia), 40 µg/mL gentamicin, and 250 mg/L glutamine. The matrices were investigated during static cultivation in a 12-well plate. The cytocompatibility of the samples was assessed using the live/dead cell staining. Live and dead cells were visualized using a Zeiss LSM 750 confocal laser-scanning microscope (Carl Zeiss Microscopy GmbH, Jena, Germany). For cell fluorescence imaging, double staining with acridine orange (green) and ethidium bromide (red) was used. Live cells were stained with acridine orange, and dead cells were stained with ethidium bromide. The quantitative indicator of the cell cultures’ viability was evaluated using a colorimetric MTT assay. The MTT (3-(4,5-dimethylthiazol-2-yl)2,5-diphenyl tetrazolium bromide) is a popular tool in estimating the metabolic activity of living cells. A quantitative indicator of viability is the coefficient of the decrease in viable cells within 24 h per unit volume of suspension with cell culture under cytotoxic effect. For a cytotoxic test, freshly isolated cells’ well plates were incubated together with the samples. Viable cells were counted relative to the initial number of cells using the microscope in the Goryaev chamber.

To carry out the hemolysis test, healthy donor blood containing sodium citrate (3.8 wt.%) in a ratio of 9:1 was diluted with normal saline (4:5 ratio by volume). The erythrocyte hemolysis test shows the interaction of the entire surface of the biomaterial with blood cells, which are necessary for the oxygen transfer by the blood to tissue cells and promoting their oxidative processes. Intact and modified TiNi samples were dipped into a standard tube containing 10 mL of normal saline that had been previously incubated at 37 °C for 30 min. Next, 0.2 mL of diluted blood was added to the standard tube, and the mixtures were incubated for 60 min at 37 °C. Similarly, normal saline solution was used as a negative control and deionized water was used as a positive control. After that, all of the tubes were centrifuged for 5 min at 3000 rpm, and the supernatant was carefully removed and transferred to a cuvette for spectroscopic analysis at 545 nm. In addition, hemolysis was calculated using a Uniplan ultraviolet spectrophotometer (Pikon Inc., RF, Tokyo, Japan). Hemolysis percent was the average of three replicates, which was calculated as follows:

$$\mathrm{Hemolysis}\;=\frac{\left(\mathrm{OD}\;\left(\mathrm{test}\;\mathrm{sample}\right)-\mathrm{OD}\;\left(\mathrm{negative}\;\mathrm{control}\right)\right)\;}{(\left(\mathrm{OD}\;\left(\mathrm{positive}\;\mathrm{control}\right)-\mathrm{OD}\;\left(\mathrm{negative}\;\mathrm{control}\right)\right)}\times 100\%$$

## 3. Results and Discussion

**Figure 1:**
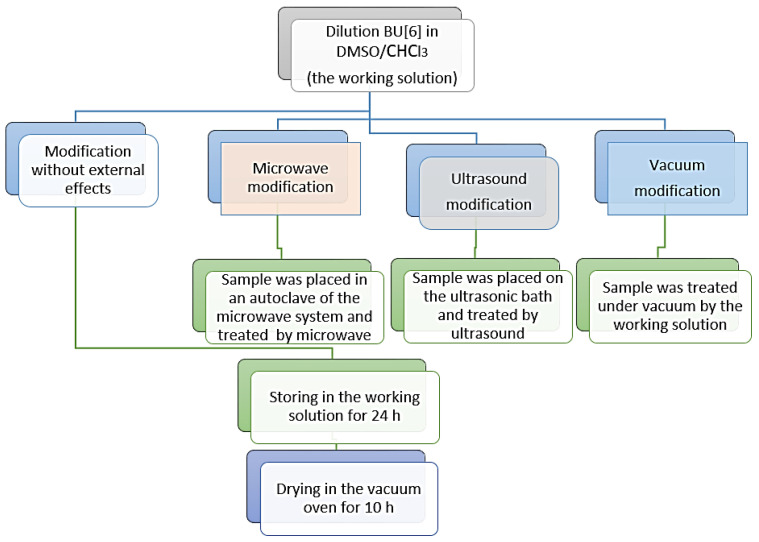
Flow chart of BU[6] deposition on porous TiNi samples.

BU[6] was deposited on the porous TiNi surface from solution under different physical effects of vacuum, microwaves, ultrasound and without external effects (Figure 1).

Modification of porous TiNi caused an intermolecular interaction between BU[6] and the TiNi surface due to the developed surface morphology, dendritic structure of BU[6] agglomerates, and electrostatic interaction. Donor–acceptor interaction is widespread due to the charge transfer between donor and acceptor molecules with no chemical bond formed (exchange mechanism), which is characteristic of the formation of compounds of d-elements.

**Figure 2:**
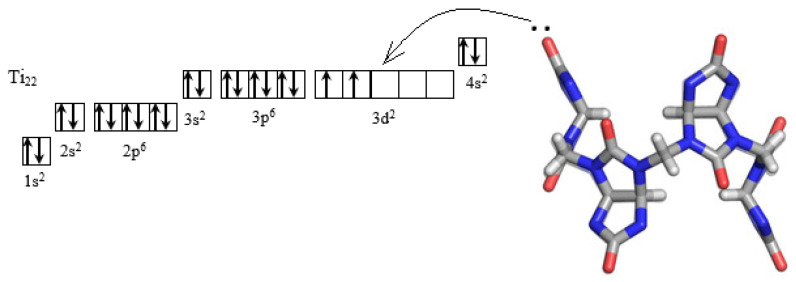
Scheme of donor–acceptor interaction between BU[6] and Ti_22_.

Theoretical calculations of bambusuril[6] and its electrostatic potential map were obtained by Tomas Lizal and Vladimir Sindelar [19]. The electrostatic potential indicates that the BU[6] cavity is the most positive region, with carbonyl oxygen atoms forming the most negative region [19,31]. Two lone pairs of electrons are localized on the carbonyl oxygen of BU[6]. The electronic configuration of titanium comprises three vacant d-orbitals, which indicates that the lone electron pair is transferred from oxygen to titanium, providing a donor–acceptor interaction between BU[6] and the TiNi surface (Figure 2).

### 3.1. SEM Study

**Figure 3:**
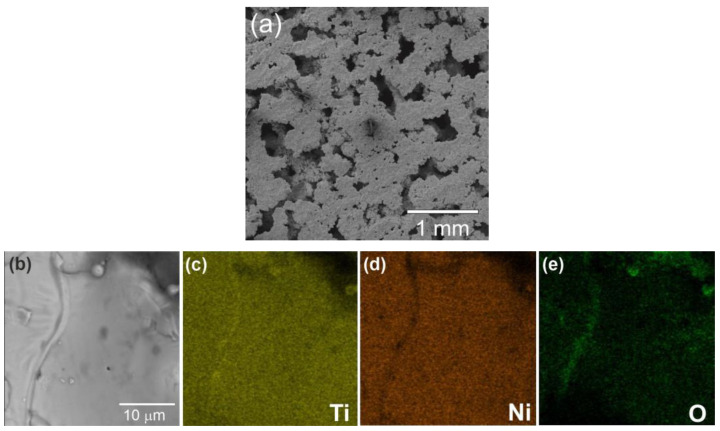
General view of control porous TiNi sample (**a**), SEM image of the control TiNi sample surface at different magnifications (**a**,**b**) with EDS maps of the element composition (**c**–**e**).

Figure 3a shows a general view of the microstructure of the control porous titanium nickelide. The EDS mapping of the surface element composition of the control porous TiNi surface layer has revealed the presence of Ti, Ni, and O content (Figure 3b–e). The formation process of the NiTi intermetallic compound matrix, surface layer, and structure is presented in detail in [3].

**Figure 4:**
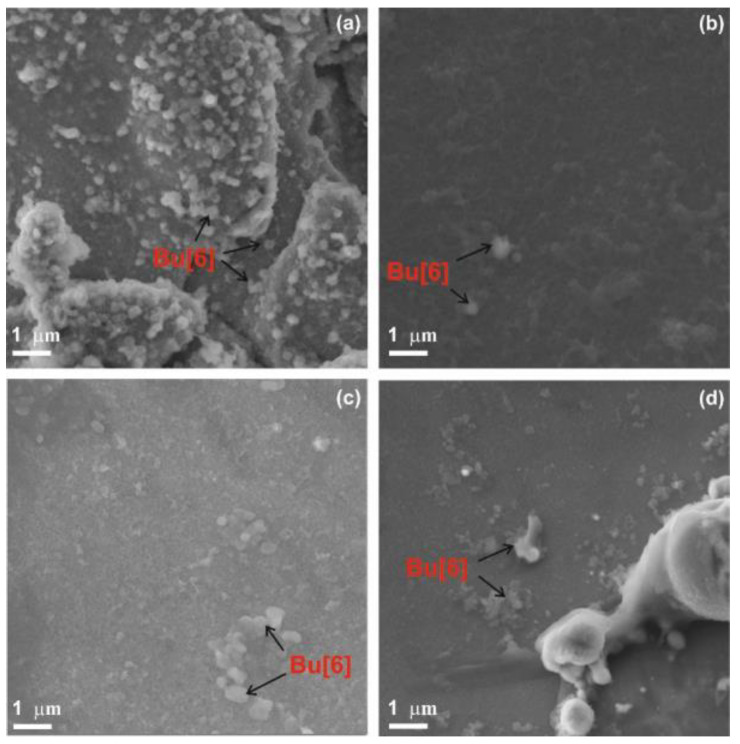
SEM images of the surface of porous TiNi with bambusuril deposited under vacuum (**a**); without external effects (**b**); under microwave exposure (**c**); under ultrasonic exposure (**d**).

**Figure 5:**
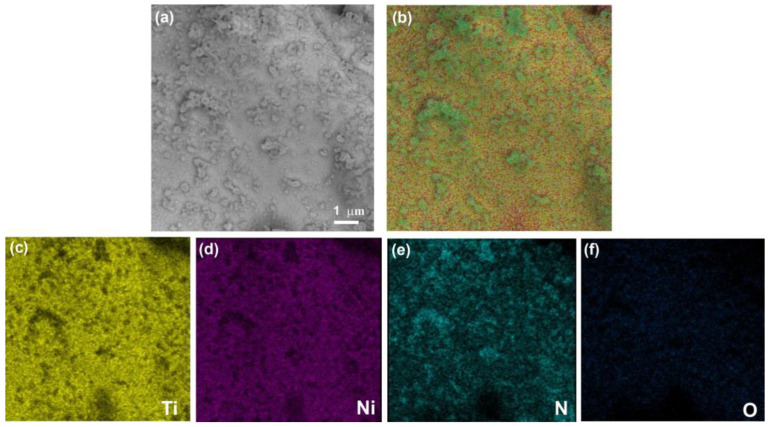
SEM images (**a**) and EDS layered image (**b**) of the surface morphology of the porous TiNi with bambusuril deposited under vacuum with distribution maps of Ti (**c**), Ni (**d**), N (**e**), and O (**f**) elements.

The morphology of the BU[6]-modified TiNi surface was studied using SEM images (Figure 4). The method of bambusuril deposition directly affects the size of agglomerated particles and the distribution frequency. The bambusuril content is close to the detection limit of EDS spectroscopy. Its presence was evidenced by the nitrogen content in the EDS results (Figure 5, Table 1). BU[6] agglomerates of a predominantly spherical shape deposit on the TiNi surface in an island-like manner; the size of the agglomerated particles varies from 0.3 to 1 μm. Under vacuum (Figure 4a), BU[6] agglomerates densely and uniformly cover the outer and inner surfaces of the pores, while other methods enable deposition of single or small bambusuril agglomerates on the outer surface of the pores. Inside the pores, a sufficient amount of bambusuril was found only under vacuum conditions.

### 3.2. Thermogravimetric (TG) and Derivative Thermogravimetric (DTG) Curves

**Figure 6:**
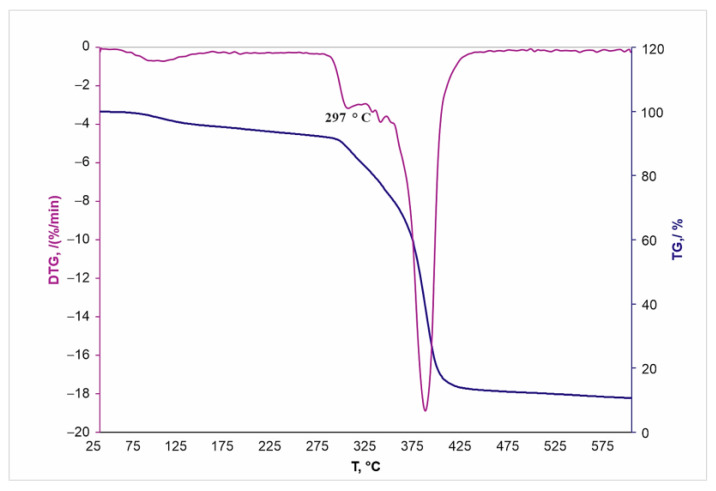
TG and DTG for BU[6].

**Figure 7:**
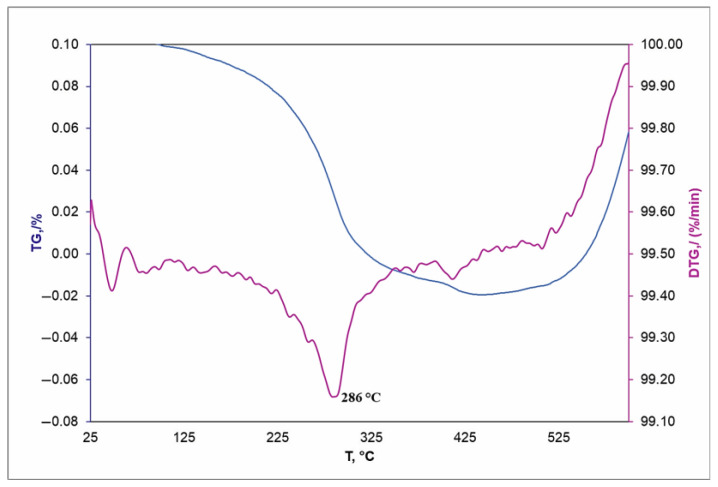
TG and DTG of TiNi modified with BU[6] under vacuum.

Since the vacuum modification method provides a uniform and dense deposition of BU[6] on the surface, including the inner pore surface, a thermogravimetric analysis was carried out for these samples. This method detects BU[6]. Figure 6 and Figure 7 show thermogravimetric (TG) curves and derivatives of the thermogravimetric (DTG) curves of BU[6] and BU[6]-modified TiNi. The percentage of weight loss in the modified sample after heating to 600 °C attained 0.2%, which indicates the amount of BU[6] on the porous TiNi surface.

The melting temperature of BU[6] attained 297 °C (Figure 6), while that of BU[6] bound with the porous TiNi surface was 286 °C (Figure 7). The melting temperature of BU[6] after its deposition on the porous TiNi surface decreases due to the following: an increased effect of surface energy, namely, its increased contribution to free energy, decreases the melting temperature of BU[6] on the TiNi surface as the particle size reduces, since the surface energy of the liquid phase at its interface with vapor is lower than that of the solid phase.

### 3.3. In Vitro Test

To study the effect of BU[6] deposition methods on cell survival, the MTT test was performed in vitro, and the percentage of hemolysis was calculated for all the modified alloys. The hemolytic index for the control sample of porous TiNi was 0.6 ± 0.1%. The percentage of erythrocyte hemolysis was 0.3 ± 0.1% under vacuum, 1.4 ± 0.2% under microwave exposure, and 1.6 ± 0.2% under ultrasonic exposure (Table 2). The calculated hemolytic index for these methods did not exceed 2% and corresponded to the normal hemolytic index for biomaterials in contact with blood (ISO 10993-4: 2020), which indicates the feasibility of these methods for surface modification of biomaterials. Without external effects, the hemolytic index of the method was 6.5 ± 0.2% and exceeded by 10.8 fold that of the control sample. This is due to a large amount of the organic solvent (DMSO:CHCl3) on the TiNi surface, which is used for BU[6] solvation.

**Figure 8:**
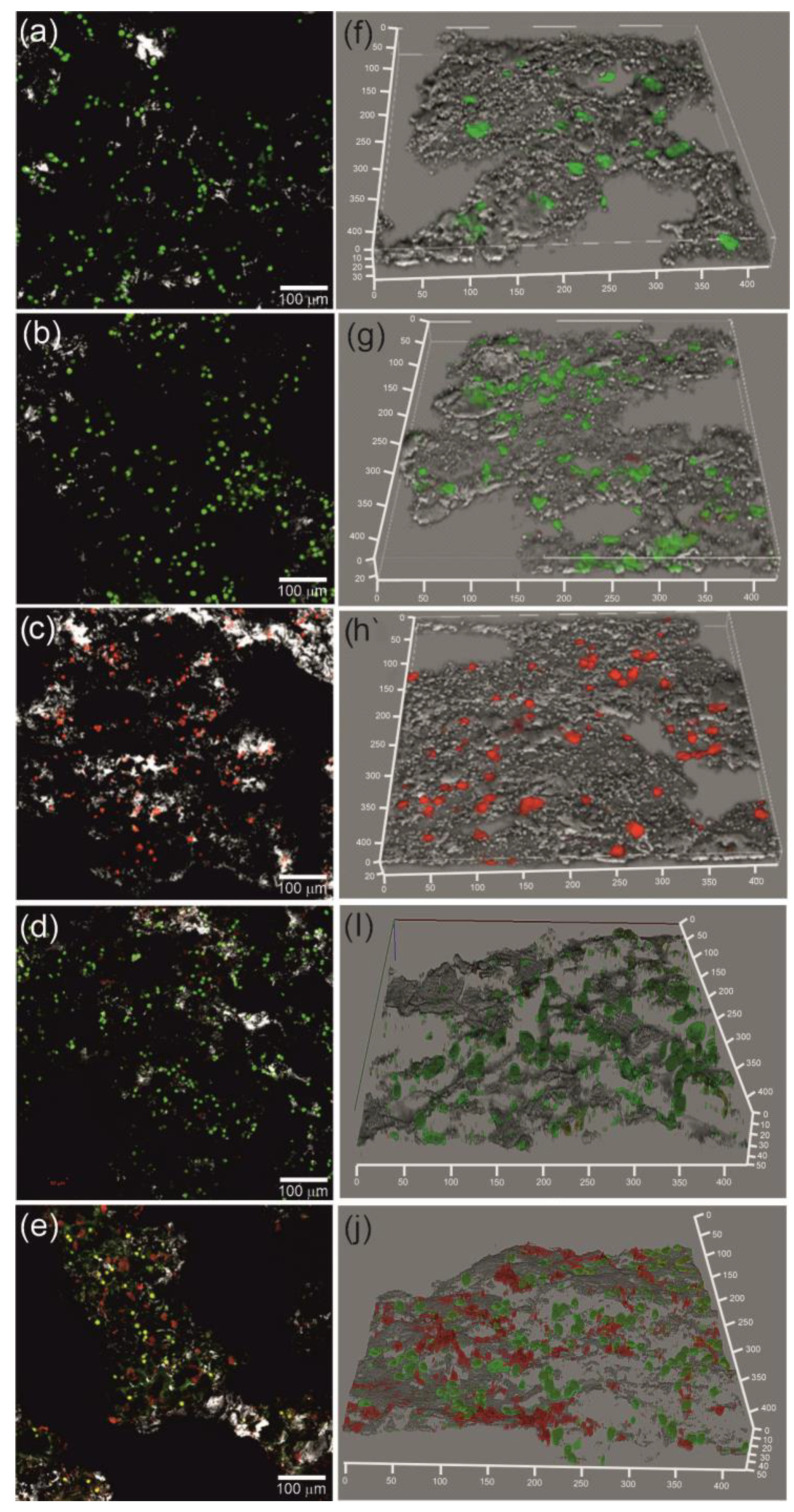
Confocal laser scanning microscopy of porous samples after 24-h culturing with MCF-7 cells: control sample (**a**,**f**), vacuum modification method (**b**,**g**), without external effects (**c**,**h**), microwave modification method (**d**,**i**), ultrasonic modification method (**e**,**j**).

The MTT test is one of the most sensitive methods for evaluating the cytotoxicity of biomaterials. We employed the MTT test to study the metabolic activity of cells on the porous TiNi surface with deposited BU[6] molecules. The quantitative ratio of live/dead cells on the porous TiNi surface varied depending on the deposition method used. Figure 8 shows confocal laser scanning microscopy of TiNi-based samples after 24-h culturing with MCF-7 cells.

**Figure 9:**
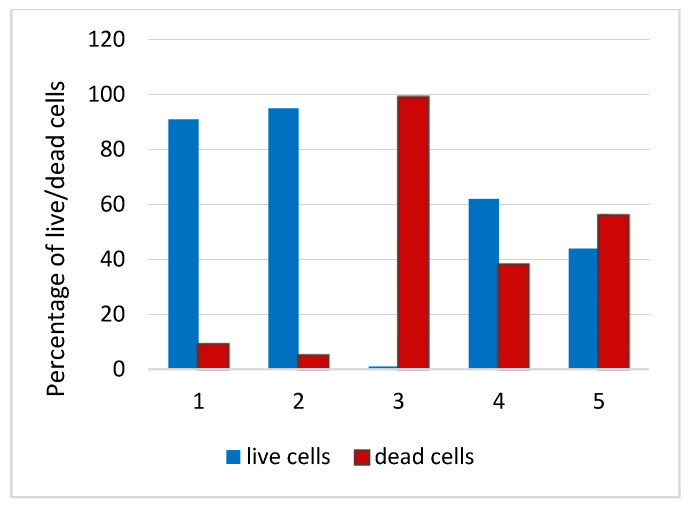
Percentage of live/dead cells after 24-h culturing with MCF-7 cells of TiNi samples treated with BU[6] by different methods: 1—control sample, 2—vacuum modification method, 3—without external effects, 4—microwave modification method, 5—ultrasonic modification method.

The control sample and the sample with the surface modified under vacuum exhibited enhanced surface cytocompatibility. The percentage of live cells in these samples exceeded 90% (Figure 9). It should be noted that the sample modified without external effects exhibited the worst biocompatibility. The percentage of live cells was only 0.7%, which may be due to the fact that DMSO used to dissolve BU[6] significantly changes the morphology and attachment of cells. In this case, the residual solvent on the surface reduces cell viability. The microwave and ultrasonic methods caused partial cell death, and the percentage of live cells was 62% and 44%, respectively. The vacuum modification method had better biocompatibility in vitro, since it contained more BU[6] on its surface, which provides better cytocompatibility and decreases erythrocyte hemolysis.

## 4. Conclusions

Based on structural data, NMR spectroscopy, and thermogravimetric analysis, a novel method has been developed for functionalization of the surface of porous TiNi alloys with BU[6]. The thermogravimetric and NMR methods of analysis, and the nitrogen content based on EDS results, made it possible to identify the presence of BU[6]. The SEM study of the structure of the modified TiNi surface with BU[6] under vacuum showed that BU[6] agglomerates 0.3–1 μm in size are uniformly deposited on the inner and outer surfaces of TiNi pores. It will provide an even saturation of BU[6] cavities with various pharmaceuticals, including antibiotics and inhibitors. The ultrasonic and microwave methods, and the method without external effects, locally yield BU[6] islands on the sample surface, but not inside the pores.

In vitro tests proved high biocompatibility and low toxicity of porous TiNi treated with BU[6] under vacuum. The control sample and the sample with the surface modified under vacuum exhibited enhanced surface cytocompatibility. The percentage of live cells in these samples exceeded 90%. The hemolytic index for the control sample of porous TiNi was 0.6 ± 0.1%, and for the modified sample under vacuum it was 0.3 ± 0.1%. The calculated hemolytic index corresponds to the normal hemolytic index for biomaterials in contact with blood.

Thus, vacuum deposition of macrocyclic compounds on the surface of porous implants will enable development of advanced materials with an appropriate micro-environment for osteogenesis and controlled release of bioactive substances, for example, antibacterial agents. Moreover, a contact interaction with osteogenic cells will not cause material encapsulation, thus ensuring rapid reparative osteogenesis.

## Figures and Tables

**Table 1:** SEM-EDS of the porous TiNi with bambusuril deposited under vacuum.

Elements	Ti (±2)	Ni (±2)	N (±1)	O (±1)
wt.%	42	34	9	15

**Table 2:** Percentage of hemolysis for samples modified with BU[6] by different methods on the porous TiNi surface.

Modification Method	Control TiNi Sample	Under Vacuum	Without External Effects	Under Microwave Exposure	Under Ultrasonic Exposure
Hemolysis (%)	0.6	0.3	6.5	1.4	1.6

## Data Availability

Not applicable.
